# ISOLATION OF BACTERIA CAUSING SECONDARY BACTERIAL INFECTION IN THE LESIONS OF CUTANEOUS LEISHMANIASIS

**DOI:** 10.4103/0019-5154.43217

**Published:** 2008

**Authors:** Hengameh Ziaie, G Sadeghian

**Affiliations:** *From Medical School of Isfahan University of Medical Science, Isfahan, Iran*; 1 *From Skin Disease and Leishmaniasis Research Centre (SDLRC), Isfahan University of Medical Science, Isfahan, Iran*

**Keywords:** *Bacteria*, *cutaneous leishmaniasis*, *infection*

## Abstract

**Background:**

Cutaneous Leishmaniasis (CL) is a parasitic disease characterized by single or multiple ulcerations. Secondary bacterial infection is one of the complications of the disease that can increase the tissue destruction and the resulting scar.

**Objective:**

To effectively determine the incidence of real secondary bacteria infection in cutaneous leishmaniasis, we designed the current study.

**Methods and Materials:**

This was a cross-sectional study performed in Skin Diseases and Leishmaniasis Research Centre, Isfahan, Iran. In this study, 854 patients with confirmed CL were enrolled. Samples were taken from all the patients. Sterile swaps were achieved for the ulcer exudates and scraping was used for nonulcerated lesions. All the samples were transferred to tryptic soy broth medium. After 24 h of incubation in 37°C, they were transferred to eosin methylene blue agar (EBM) and blood agar. Laboratory tests were used to determine the species of bacteria. All of the collected data were analyzed by SPSS software and chi-square.

**Results:**

Among 854 patients with confirmed cutaneous leishmaniasis, 177 patients (20.7%) had positive cultures for secondary bacterial infection. Bacteria isolated from the lesions were as follows: *Staphylococcus aureus* - 123 cases (69.4%), coagulase negative *Staphylococcus* - 41 cases (23.1%), *E. coil* - 7 cases (3.9%), *Proteus* - 3 cases (1.7%) and *Klebsiella* - 3 cases (1.7%).

**Conclusions:**

The incidence of secondary bacterial infection in lesions of CL was 20.7%. The most common isolated pathogen was *Staphylococcus aureus*. The incidence of secondary bacterial infection was significantly more in the ulcerated lesions as compared with nonulcerated lesions (*P* = 0.00001).

## Introduction

Leishmaniasis is a parasitic disease transmitted by sand flies. It is characterised by a spectrum of cutaneous, mucocutaneous and visceral clinical manifestations that depend largely on the species of parasite involved and the host immune response. According to recent estimates, 1.5 million new cases of cutaneous leishmaniasis (CL) occur each year. More than 90% of cases occur in five countries in the Old World (Afghanistan, Algeria, Iran, Iraq and Saudi Arabia) and two countries in the New World (Brazil and Peru).^1^ Cutaneous leishmaniasis in the Old World is caused by *L major*, *L. tropica*, *L infantum* and *L. aethiopica*, which are found in southern Europe, the Mediterranean basin, the Middle-East and Africa.^2^ Cutaneous leishmaniasis in the New World is mainly caused by members of the *L. braziliensis* complex (*L. braziliensis* and *L. peruviana*), *L. mexicana*, *L. amazonensis* and the *L. guyanensis* complex (*L. guyanensis* and *L. panamensis*).

Cutaneous leishmaniasis of the Old World eventually heals. The rate of spontaneous healing depends on several factors, including parasite load and virulence, host immune response, location of the lesion and the presence or absence of secondary bacterial infection. Lesions caused by *L. major* heal spontaneously after approximately 18 weeks.^3^

An important part of therapy for CL is local care along with antileishmania therapy. The treatment of secondary bacterial infection is essential for healing. On the other hand, the secondary bacterial infection of the CL will increase the tissue destruction and the resulting scar.^4^ To effectively determine the incidence of real secondary bacteria infection in CL, we designed the present study.

## Materials and Methods

This was a cross-sectional study performed at Skin Disease and Leishmaniasis Research Centre (SDLRC), Isfahan, Iran.

The patients enrolled in this study with clinical and parasitological diagnosis of CL and referred to SDLRC from August 2006 to 2007. The patients belonged to both sex and different age groups and had different clinical form of CL. The use of topical or systemic antibiotics in recent weeks was not included in the study.

The skin areas surrounding the lesions were thoroughly cleaned using cotton wool moistened with alcoholic iodine. After appropriate cleaning, the specimens of the ulcers were obtained by rubbing sterile saline solution over the edge of ulcerated lesions and samples were collected aseptically by scraping the nonulcerated lesions. All the samples were transferred to tryptic soy broth medium. After 24-h incubation in 37°C, the samples were transferred to eosin methylene blue agar (EMB) and blood agar.

Gram staining, oxidase test, indole test, urease test, catalase test and coagulase test were used to determine the species of bacteria.

### Statistical analysis

All of the collected data were analysed by SPSS and Chi-squared test.

## Results

In this study, 854 patients who confirmed CL were enrolled. The age range of patients was 2 months to 85 years and the mean age of the patients was 27.21 years. Among them, 320 patients (37.5%) were women and 534 (62.5%) were men. The results of the culture were positive in 20.7% of the patients (177 patients). Bacteria isolated from the lesions were as follows: *Staphylococcus aureus* - 123 cases (69.4%), coagulase negative *Staphylococcus* - 41 cases (23.1%), *E. coli* - 7 cases (3.9%), *Proteus* - 3 cases (1.7%) and *Klebsiella* - 3 cases (1.7%).

The distribution frequency of the isolated bacteria by the clinical from of the leishmaniasis lesions are shown in Table 1.

**Table 1:** Distribution frequency of bacteria in positive cultures

Clinical forms of lesion	Ulcerated plaque	Nonulcerated plaque	Ulcerated papule or nodule	Total
*Staphylococcus aureus*	117 (66.1)	3 (1.7)	3 (1.7)	123 (69.4)
Coagulase negative *Staphylococcus*	41 (23.1)	-	-	41 (23.1)
*E. coli*	7 (3.9)	-	-	7 (3.9)
Proteus	3 (1.7)	-	-	3 (1.7)
Klebsiella	3 (1.7)	-	-	3 (1.7)
Total positive culture	171 (96.6)	3 (1.7)	3 (1.7)	177 (100)

Figures in parenthesis are in percentage

## Discussion

Secondary bacterial infection is one of the complications of CL. Although some authors emphasize on the rarity of this finding,^3^ our clinical findings are in contrast to this. In our practice, we encounter many cases of the infected leishmaniasis ulcers. In fact, secondary bacterial infection can exacerbate the disease and the final scar because it will increase the tissue destruction and necrosis. In these cases, painful ulcers with purulent discharges and with surrounding inflammation may occur. In addition, the duration of disease would be prolonged.^4^ The appropriate use of antibiotics will decrease the resultant infection in these cases. One study in Sudan has shown the prevalence of the secondary infection to be 18% of the 736 evaluated patients. The pathogenic organism was not identified in this study.^5^

In the another study, bacteria including *Proteus vulgaris*, *Pasteurella* multisided, *Staphylococcus aureus*, *Staphylococcus albus*, *E. coli* and *Pseudomonas aeruginosa* were isolated from clinically infected lesions.^6^

In another study that was performed in the impetiginized forms of leishmaniasis, *Staphylococcus aureus* was recognized to be the responsible pathogen.^4^

In a study performed in the Yucantan peninsula of Mexico, some pathogenic bacteria were detected from the skin lesions of patients with chiclero's ulcers (a form of cutaneous leishmaniasis due to *Leishmania mexicana*) reluctant to antimonial treatment, and the results of this study suggested the need to eliminate bacterial infections by antibiotic therapy before starting antimonial administration.^7^

In our study, out of the 854 patients with confirmed cutaneous leishmaniasis, 177 patients (20.7%) had confirmed secondary bacterial infection.

The most common bacterial isolate was *Staphylococcus aureus*. Other pathogens included coagulase negative *Staphylococcus*, *E. coli*, *Proteus vulgaris* and *Klebsiella*.

There was no significant association between the prevalence distribution of the isolated bacteria with the age, sex, location, number and duration of the disease (*P* > 0.05). However, there was a significant association between the clinical form of the disease and the isolated bacteria (*P* = 0.00001).

All the lesions that had secondary bacterial infection were ulcerated. There was no bacterial isolates from the lesions that were not ulcerated. With regard to these facts, we can conclude that destruction of the epidermis in the ulcerated lesions had predisposed the patients to the secondary bacterial infection.

Regarding the results of our study, we suggest that topical antiseptic solutions are need for ulcerated lesions of the cutaneous leishmaniasis to prevent the secondary bacterial infection that may accelerate tissue destruction.

In addition in the case of secondary bacterial infection symptoms and signs, the use of antibiotics particulary against staphylococcus would be logical.
